# ViVaMBC: estimating viral sequence variation in complex populations from illumina deep-sequencing data using model-based clustering

**DOI:** 10.1186/s12859-015-0458-7

**Published:** 2015-02-22

**Authors:** Bie Verbist, Lieven Clement, Joke Reumers, Kim Thys, Alexander Vapirev, Willem Talloen, Yves Wetzels, Joris Meys, Jeroen Aerssens, Luc Bijnens, Olivier Thas

**Affiliations:** 1 Department of Mathematical Modeling, Statistics and Bioinformatics, Ghent University, Coupure Links 653, Gent, 9000 Belgium; 2 Department of Applied Mathematics, Informatics and Statistics, Ghent University, Krijgslaan 281 S9, Gent, 9000 Belgium; 3 Janssen R&D, Janssen Pharmaceutical Companies of J&J, Turnhoutseweg 30, Beerse, 2340 Belgium; 4 ExaScience Life Lab, Kapeldreef 75, Leuven, 3001 Belgium; 5 University of Wollongong, National Institute for Applied Statistics Research Australia (NIASRA), School of Mathematics and Applied Statistics, NSW, 2522 Australia

**Keywords:** Illumina sequencing, Codon, Second best base call, Model-based clustering, Viral quasispecies

## Abstract

**Background:**

Deep-sequencing allows for an in-depth characterization of sequence variation in complex populations. However, technology associated errors may impede a powerful assessment of low-frequency mutations. Fortunately, base calls are complemented with quality scores which are derived from a quadruplet of intensities, one channel for each nucleotide type for Illumina sequencing. The highest intensity of the four channels determines the base that is called. Mismatch bases can often be corrected by the second best base, i.e. the base with the second highest intensity in the quadruplet. A virus variant model-based clustering method, ViVaMBC, is presented that explores quality scores and second best base calls for identifying and quantifying viral variants. ViVaMBC is optimized to call variants at the codon level (nucleotide triplets) which enables immediate biological interpretation of the variants with respect to their antiviral drug responses.

**Results:**

Using mixtures of HCV plasmids we show that our method accurately estimates frequencies down to 0.5%. The estimates are unbiased when average coverages of 25,000 are reached. A comparison with the SNP-callers V-Phaser2, ShoRAH, and LoFreq shows that ViVaMBC has a superb sensitivity and specificity for variants with frequencies above 0.4%. Unlike the competitors, ViVaMBC reports a higher number of false-positive findings with frequencies below 0.4% which might partially originate from picking up artificial variants introduced by errors in the sample and library preparation step.

**Conclusions:**

ViVaMBC is the first method to call viral variants directly at the codon level. The strength of the approach lies in modeling the error probabilities based on the quality scores. Although the use of second best base calls appeared very promising in our data exploration phase, their utility was limited. They provided a slight increase in sensitivity, which however does not warrant the additional computational cost of running the offline base caller. Apparently a lot of information is already contained in the quality scores enabling the model based clustering procedure to adjust the majority of the sequencing errors. Overall the sensitivity of ViVaMBC is such that technical constraints like PCR errors start to form the bottleneck for low frequency variant detection.

**Electronic supplementary material:**

The online version of this article (doi:10.1186/s12859-015-0458-7) contains supplementary material, which is available to authorized users.

## Background

In a virology research environment, the study of viral quasispecies in infected patients is essential for understanding pathways to resistance and can substantially improve treatment. Genotypic and phenotypic methods are commonly used for detecting antiviral resistance in clinical HIV-1 and HCV specimens. Standard genotyping such as direct PCR sequencing methods, however, only provides information on the most abundant sequence variants. Modern massive parallel sequencing (MPS) technologies, on the contrary, have the opportunity to allow in-depth characterization of sequence variation in more complex populations, including low-frequency viral strains. However, one of the challenges in the detection of low-frequency viral strains concerns the errors introduced during the sequencing process. As these specific errors may occur at equal or even higher frequencies than true biological mutations, a powerful assessment of low-frequency virus mutations is seriously jeopardized [1,2].

Many proposals have been made to address this challenge of decreased detection power. Several authors compared the distribution of variants to Poisson, binomial or beta-binomial error distributions [3-7]. They all, however, assume that base calls are of equal quality which is not the case in MPS [8,9]. As a potential solution other authors suggested to incorporate quality scores when modeling the error distribution [10-13]. Many of these methods focus primarily on 454 data [3-6,11,13]. The announcement by Roche to fade out the 454 technology by mid 2016, illustrates the pressing need to focus on alternative technologies [14]. Moreover, the incorporation of quality scores is most appropriate for Illumina sequencing data. Illumina quality scores reflect the base calling substitution error probabilities [15], whereas 454 quality scores do not have such an intuitive interpretation [16]: they represent the probability of calling a homopolymer up to a particular length.

Illumina’s sequencing technology is a sequencing-by-synthesis technology where the DNA fragments are synthesized one base at a time. The DNA fragments to be sequenced are first spatially separated and amplified, resulting in clusters of identical sequences on the sequencing flow cell. Identification of different bases in the sequencing-by-synthesis process is enabled by using distinct fluorophores for each nucleotide type (A,C,T,G). At every sequencing cycle a single labeled 3’-blocked nucleotide is incorporated to the complementary strand of each DNA fragment. The fluorophore is determined with imaging technology using four different fluorescence channels, one for each nucleotide type. For every fragment in each cycle, the base caller assigns the nucleotide that corresponds with the highest intensity among the four channels. A correct base identification is complicated by multiple effects. On the one hand, emission spectra of the fluorophores are overlapping, especially the A and C intensities and the G and T intensities. On the other hand, phasing and pre-phasing describes the loss of synchrony of the sequence copies of a cluster. Phasing is caused by incomplete removal of the 3 ^′^-protecting groups resulting in sequences within clusters lagging behind in the incorporation cycle. Pre-phasing is caused by the incorporation of nucleotides without effective 3 ^′^-protecting groups. This can cause incorporation of multiple bases in each cycle and might hamper a correct interpretation of the intensities. Quality scores are derived from the intensities [17]. From literature [18] and own experiments it is clear that these quality scores often underestimate the true error probabilities. Extra information which can be used in this context is the second best base calls, which are the bases corresponding to the second highest intensity. Abnizova et al. [19] observed that a mismatch base could often be corrected by its second best base call. In an experiment with known reference sequence 722,505 codons were evaluated of which 34,644 were errors (≈5%). Seventy percent of these errors could be corrected by the second best base calls (see later for more details). Hence, we will explore the utility of second best base calls in addition to the quality scores within a new variant calling algorithm.

Here we propose Virus Variant Model-Based Clustering (ViVaMBC), a method that models error probabilities of the best and second best base calls as a function of the Illumina quality scores. These error probabilities are embedded in a multinomial mixture so that viral variants can be identified and quantified. This paper will illustrate and validate this method using read sets with known variation and evaluate the minimum sequencing depth. Its performance will be empirically compared with three other methods (LoFreq [10], V-Phaser2 [12] and ShoRAh [5]). Finally, we will demonstrate ViVaMBC on a clinical HCV sample.

## Methods

### Experiments

Several samples from HCV-infected patients as well as HCV plasmids were paired-end sequenced using Illumina’s genome analyzer(GA)IIx according to manufacturing protocols. A detailed description of the data and protocols is given in Thys et al. (Thys K, Verhasselt P, Reumers J, Verbist BMP, Maes B, Aerssens J. Performance assessment of the Illumina massively parallel sequencing platform for deep sequencing analysis of viral minority variants, submitted). The sequencing images are converted into reads using Illumina’s off-line base caller (OLB) [20]. In contrast to the standard workflow, using real time analysis (RTA), the OLB can also provide second best base calls which are explored for an improved error correction. In the next steps reads are aligned against a consensus sequence using BWA [21]. The resulting bam files are adapted using GATK clipReads to prevent trimming of the data, and all reads containing indel errors are removed (workflow presented in Additional file 1). It is hereby assumed that indels will result in non-viable viruses. These bam files are used as input of ViVaMBC which is explained in the next section.

### Model-based clustering

Let **r**_*i*_ denote the vector with the best base calls of read *i*, with *i* ranging from 1 to n. Similarly, **s**_*i*_ denotes the vector with the second best base calls of read *i*. The vector with the corresponding error probabilities is denoted as **θ**_*ri*_ and **θ**_*si*_ for best and second best base calls respectively. A dummy variable Pair _*i*_ is introduced to indicate which end of the DNA segment is sequenced in the paired-end sequencing strategy: *P* *a* *i* *r*_*i*_ equals 1 if read *i* is first in pair and 0 otherwise. In case of single-end experiments the variable *P* *a* *i* *r*_*i*_ can simply be omitted from the model. The library of reads represent the whole viral population consisting of several viral subspecies.

The variant calling is applied locally. Upon read alignment the vectors **r**_*i*_ are retained that cover a small window of the reference sequence under investigation. To avoid the challenges involved in inferring haplotypes beyond the actual read lengths [22], only windows smaller than the read length are considered. Let *m* denote the length of the window; thus $\mathbf {r}_{i}^{t}=(r_{i1}, \ldots, r_{\textit {im}})$, *A* *P*_*i*_ denote the average quality score of read *i* in window *m* and **θ**_*oil*_ with *l*=(1,…,*m*) denotes the probability that the *l*th nucleotide from read *i* differs from *r*_*il*_ or *s*_*il*_.

Suppose that *k* variants of length *m* exist with variant sequences given by the vectors **h**_1_,…,**h**_*k*_. Let *τ*_*j*_ denote the prior probability that a read originates from variant *j* (*j*=1,…,*k*). They have the interpretation of relative frequencies of the viral variants within the window, which are the key parameters of interest inferred from the observed data.

The likelihood of the observed data has the natural interpretation of a mixture model with *k* components that refer to the true variants. The likelihood is the product of the probabilities that a read was generated from the mixture of variants with relative frequencies *τ*_*j*_:

(1) $$ L = \prod\limits_{i=1}^{n} f(\mathbf{r}_{i},\mathbf{s}_{i}) = \prod\limits_{i=1}^{n} \left[\sum\limits_{j=1}^{k} \tau_{j}\, f_{j}(\mathbf{r}_{i},\mathbf{s}_{i})\right],  $$

where *f* denotes a generic density function and *f*_*j*_ is the probability of observing best calls **r**_*i*_ and second best calls **s**_*i*_ when read *i* belongs to variant *j*. Upon relying on the multinomial distribution, the probability *f*_*j*_ can be written as

(2) $${} \begin{aligned} f_{j}(\mathbf{r}_{i},\mathbf{s}_{i}) &= \prod\limits_{l=1}^{m} f_{j}(r_{il},s_{il}) \\&= \prod\limits_{l=1}^{m} \theta_{ril}^{I\left({r_{il}=h_{jl}}\right)} \theta_{sil}^{I\left({s_{il}=h_{jl}}\right)} \theta_{oil}^{\left(1-I\left({r_{il}=h_{jl}}\right)\right)\left(1-I\left({s_{il}=h_{jl}}\right)\right)}, \end{aligned}   $$

in which *I*(*A*)=1 if *A* is true, and *I*(*A*)=0 otherwise. Note, however, that the probabilities *θ*_*ril*_, *θ*_*sil*_ and *θ*_*oil*_ can not be estimated from the data because the model is over-identified (two parameters for each observation). We therefore model the *θ* parameters as a function of the quality scores of the best base calls (*P*_*ril*_), a dummy variable Pair (*P* *a* *i* *r*_*i*_), and the average quality score (*A* *P*_*i*_). For each location *l*, the *θ*’s refer to a multinomial distribution with three classes for which we suggest a multinomial logit model

(3) $$ log \frac {\theta_{cil}} {\theta_{oil}} = \beta_{0c} + \beta_{1c}P_{ril} + \beta_{2c}{Pair}_{i} + \beta_{3c}{AP}_{i},  $$

with *c*∈{*r*,*s*}. For paired-end experiments eight *β* parameters need to be estimated together with the variant sequences *h*_*j*_ (*j*=1,…,*k*) and the relative frequencies *τ*_*j*_. For single-end experiments two *β* parameters are removed since the *P* *a* *i* *r*_*i*_ variable can be omitted. To infer the true variants and their frequencies the log likelihood

(4) $${} {\fontsize{8.2pt}{9.6pt}\selectfont{\begin{aligned} l = \sum\limits_{i=1}^{n} \log \left[\sum\limits_{j=1}^{k} \left(\tau_{j} \prod\limits_{l=1}^{m} \theta_{ril}^{I\left({r_{il}=h_{jl}}\right)} \theta_{sil}^{I\left({s_{il}=h_{jl}}\right)} \theta_{oil}^{\left(1-I\left({r_{il}=h_{jl}}\right)\right)\left(1-I\left({s_{il}=h_{jl}}\right)\right)} \right) \right], \end{aligned}}}   $$

after substituting the *θ*-parameters with (3) will be maximized. However, as closed form solutions for *τ*_*j*_, *h*_*j*_ and *β* are not available, numerical methods were implemented for direct maximization of the log likelihood (4). The EM algorithm is a popular alternative for maximizing mixture distributions [23,24]. It requires the introduction of latent or ’missing’ indicator variables *z*_*ij*_ which are 1 when read *i* belongs to variant *j* and zero otherwise. Note that **z**_*i*_=(*z*_*i*1_,…,*z*_*ik*_)^*t*^ are multinomial distributed with density *g*(**z**_*i*_) and *P*(*z*_*ij*_=1)=*τ*_*j*_. Hence the likelihood (1) can be augmented

(5) $${} {\fontsize{8.9pt}{9.6pt}\selectfont{\begin{aligned} L = \prod\limits_{i=1}^{n} f(\mathbf{r}_{i},\mathbf{s}_{i},\mathbf{z}_{i}) = \prod\limits_{i=1}^{n} f(\mathbf{r}_{i},\mathbf{s}_{i}\vert \mathbf{z}_{i}) g(\mathbf{z}_{i}) = \prod\limits_{i=1}^{n} \prod\limits_{j=1}^{k} \left(\, f_{j}(\mathbf{r}_{i},\mathbf{s}_{i}) \tau_{j}\right)^{z_{ij}}, \end{aligned}}}  $$

which in turn allows an efficient factorization by conditioning on **z**_*i*_. In particular, given $I^{(r)}_{\textit {ijl}} = I{(r_{\textit {il}}=h_{\textit {jl}})}$ and $I^{(s)}_{\textit {ijl}} = I{(s_{\textit {il}}=h_{\textit {jl}})}$, the complete data log-likelihood *l*_*c*_ can be written as

(6) $${} \begin{aligned} l_{c} &= \sum\limits_{i=1}^{n} \sum\limits_{j=1}^{k} z_{ij} \left\{\log\tau_{j} + \sum\limits_{l=1}^{m} \left[I^{(r)}_{ijl} \log\theta_{ril} + I^{(s)}_{ijl} \log\theta_{sil}\right.\right.\\&\qquad\qquad\qquad\qquad\;+ \left.\left. \left(1-I^{(r)}_{ijl}\right)\left(1-I^{(s)}_{ijl}\right)\log\theta_{oil}\right] \vphantom{\left\{\log\tau_{j} + \sum\limits_{l=1}^{m} \left[I^{(r)}_{ijl} \log\theta_{ril} + I^{(s)}_{ijl} \log\theta_{sil}\right.\right.}\right\}, \end{aligned}  $$

in which the *θ* parameters have to be substituted with (3). The EM algorithm iterates over an expectation (E) and a maximization (M) step until convergence.

1. E step: Computation of the expected complete data log-likelihood (6), given the observed data and the current parameter estimates. The solution is given by (6) with *z*_*ij*_ replaced by
  (7) $$ \widehat{z}_{ij} = E(z_{ij} \mid \mathbf{r}_{i}, \mathbf{s}_{i})=\frac{\widehat{\tau}_{j} \,f_{j}\left(\mathbf{r}_{i},\mathbf{s}_{i}|\widehat{h_{j}},\widehat{\beta}\right)}{\sum_{l=1}^{k} \widehat{\tau_{l}}\, f_{l}\left(\mathbf{r}_{i},\mathbf{s}_{i}|\widehat{h_{j}},\widehat{\beta}\right)}  $$
  where *f*_*j*_ depends on $\widehat {h_{j}}$ and the $\widehat {\beta }$ parameter estimates from the previous M-step.
2. M step: Maximization of the expected complete data log-likelihood from the E-step with respect to **τ**, **h**, and **β** parameters. This results in updated parameter estimates. In particular
    (8) $$ \widehat{\tau}_{j} = \frac{\sum_{i=1}^{n} \widehat{z}_{ij}}{n}   $$
*h*_*j*_ is the most abundant sequence among those with maximal $\widehat {z}_{\textit {ij}}$ across the variants (*j*=1,…,*k*).
**β** parameter estimates are obtained by fitting the multinomial regression model (3) using the $\widehat {z}_{\textit {ij}}$ as weights.

The EM algorithm is initialized with *k* variants (as a default *k* is set to 10). The *k* ^th^ most observed variants are taken as initial variant sequences **h**_*j*_ (*j*=1,…,*k*). These variants are updated in each M-step. A variant *j* will disappear if no sequences are attributed to cluster *j*. Upon convergence, the number of variants *k* and their final estimates of *τ*_*j*_ and *h*_*j*_ define the variant population in the window of size *m*.

The method is optimized for window size *m*=3, codon level, to retain linkage information between single-nucleotide polymorphisms. These codons facilitate the biological interpretation in the coding regions of the virus. Since resistance-associated mutations against antiviral drugs are particularly of interest, drug-target regions within viral protein coding regions will be investigated. Hence, the reported codon variants can be interpreted immediately with respect to their antiviral drug responses. ViVaMBC is implemented in R and parallelized. Each window of interest can be run on a different core, thereby speeding up the performance. Approximately, one position runs for 1 hour when coverages around 60,000 are reached and *m*=3. More information can be found in Additional file 1.

## Results

In the following sections, first the sensitivity and specificity of ViVaMBC with *m*=3 will be investigated using read sets with known variation. Subsequently, the minimum depth of coverage needed for unbiased estimates will be defined and its overall performance will be compared with three SNP-callers LoFreq [10], V-Phaser2 [12], and ShoRAH [5]. Finally, ViVaMBC will be illustrated on a clinical HCV sample where the NS3 region will be investigated to search for resistance associated mutations against NS3-4A serine protease inhibitors, telaprevir, and boceprevir [25].

### Sensitivity and specificity

Two different plasmids carrying HCV NS3 amino acids 1 to 181 were mixed in four different proportions. These plasmids differ only at codon positions 36 and 155. The mixing proportions were 1:10, 1:50, 1:100, and 1:200 (fastq files are available at the European Nucleotide Archive, accession number PRJEB5028; (see Thys K, Verhasselt P, Reumers J, Verbist BMP, Maes B, Aerssens J. Performance assessment of the Illumina massively parallel sequencing platform for deep sequencing analysis of viral minority variants, submitted for sample preparation). The mixtures were sequenced at an average coverage of 86,000. The plasmid mixture enables the quantification of true positives (variants at the two codon positions) and the assessment of the amount of errors that could be corrected by second best base calls (see Additional file 1). The sensitivity of ViVaMBC was quantified using the two variant positions. The estimated frequencies, *τ*_*j*_, of the real variants at codon positions 36 and 155 were close to the mixing proportions (Table 1), suggesting that frequencies down to 0.5% can be reliably estimated. Codons for the first 181 aminoacids of the NS3 region were called to investigate the specificity. No other variants are expected in this region besides the two variant positions, and hence only the wild type codons (with frequencies close to 100%) and the two variants should be detected. The number of codons reported by ViVaMBC were compared with the number of codons present in the raw data. In analogy with mpileup for SNP calling, a pileup table is built at the codon level where the low-quality parts of the reads are removed prior to the pileup, called trimming (see Additional file 1 for more details). The comparison with such a pileup table allows to assess the number of false-positive findings that are actually removed by ViVaMBC. The pileup resulted in far more than 10,000 codons while ViVaMBC detected only 599 to 841 codons in the same region (Table 2). This indicates that ViVaMBC removes the vast majority of false-positive findings. From the reported codons we removed the wild type codons with frequencies close to 100% together with the two variants and investigated the frequencies of the remaining false-positive findings. The maximum frequency of these errors is above 1% for the pileup and drops below 1% for ViVaMBC. The frequency distribution of the errors is presented in Additional Figure 1, which shows that the vast amount of frequencies for false positive variants in ViVaMBC is well below 0.4%. Some false-positive findings are expected in this frequency range as sample and library preparation errors are known to occur with frequencies up to 0.25% [26]. While the discovery of codon variants at 0.5% and 1% was hampered in the pileup table, it could be detected with almost 100% specificity using ViVaMBC. The specific contribution of the second best base error probabilities in ViVaMBC to these increased sensitivity and specificity is further explored in Additional file 1.

**Table 1:** **Sensitivity of ViVaMBC in plasmid experiment**

**Mixing prop**	**36 ATG (%)**	**155 AAA (%)**
1:200	0.45	0.42
1:100	0.92	0.91
1:50	2.28	2.20
1:10	11.04	10.01

Two HCV-plasmids which differ at two codon positions 36 and 155 were combined in a sample for Illumina deep sequencing at four different mixing proportions. Their frequencies were estimated with ViVaMBC, which was able to retrieve codon variants with frequencies up to 0.5%.

**Table 2:** **Specificity of ViVaMBC in plasmid experiment**

	**Pileup**		**ViVaMBC**	
**Mixing prop**	***N*** **° codons**	**Max noise freq (%)**	***N*** **° codons**	**Max noise freq (%)**
1:200	15,692	1.46	599	0.67
1:100	14,886	1.41	599	0.68
1:50	12,724	1.47	841	0.72
1:10	22,405	1.53	492	0.65

The number of codons in the NS3 are reported after pileup and ViVaMBC. Theoretically, 183(181+2) codons are expected, but far more are reported, especially when piling up the raw data. The maximum frequency of the false positive codons is presented as well. ViVaMBC is able to reduce these frequencies below 1% while they reached more than 1% after Pileup. This illustrated that ViVaMBC is able to reduce drastically the number of false-positive findings and to lower the detection limit above which 100% specificity is expected.

### Minimum depth of coverage

The influence of coverage depth on the accuracy of $\widehat {\tau _{j}}$ is investigated using the plasmid data by mixing 1:200 for codon position 155. The original data covered this position 64,668 times. Datasets with lower coverages are generated by random sampling a fraction (f=0.1, 0.2, …, 0.8,0.9) of the reads from the original dataset. Ten datasets were generated for each fraction *f* resulting in 90 datasets with average coverages ranging between 6,463 and 58,185.

**Figure 1:**
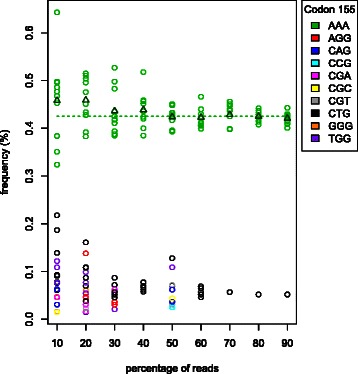
**Influence of coverage depth on the estimation of** ***τ***_*j*_ **.** Datasets with lower coverages are generated by random sampling a fraction (f = 0.1, 0.2, …, 0.8,0.9) of the reads from the original dataset. Ten datasets were generated for each fraction *f* resulting in 90 datasets with average coverages ranging between 6,463 and 58,185. The reported variants for all re-sampled datasets were plotted and colored according to the discovered codon. The green dots indicate the true variant and all others are false-positive findings. The average frequency of the true variant (averaged over the ten random samples) is indicated with triangles. The dotted line is the true frequency as estimated from the original dataset. Lowering the coverage increases the bias, the variance of the estimate and the number of false-positive findings.

ViVaMBC reported two codons for the original dataset at codon position 155: the wild type codon CGG at a frequency of 99.58% and the variant AAA at 0.42% which is indicated with the green dotted line in Figure 1. The frequencies (*τ*_*j*_) of the variants (*h*_*j*_) for this position reported by ViVaMBC for each of the 90 re-sampled datasets are plotted in Figure 1. The true codon variant AAA (green dots) was detected in all datasets. Averages frequency estimates over the 10 repeats are indicated with green triangles. Figure 1 indicates that lower coverages reduce the precision and increase the bias of the estimates. These deviations start to appear from fraction 0.4, which corresponds with coverage around 25,000. The number of false-positive findings also increases when less reads are available, but their frequency estimates remain far below 0.4% and the variant at 0.5% can still be discovered at the lowest coverage.

### Comparison with other methods

The performance of ViVaMBC is compared with LoFreq (v0.5.0) [10], V-Phaser 2 (v2.0) [12], and ShoRAH (v0.8) [5] (all ran in their default settings) using the previously described plasmid mixture data. With ShoRAH we were unable to use the original bam file since some problems were encountered when extracting the reads from the desired region. Therefore the ShoRAH results are based on a bam file with remapped reads against the reference region of interest. As none of the existing methods calls variants immediately at the codon level, the evaluation is restricted to the ability to detect variants at individual nucleotide level. The two variant codons differ at 5 nucleotides from the wild type, so 5 SNPs should be detected. The comparison is made with ViVaMBC at the codon level since these variants can be interpreted immediately with respect to their antiviral drug responses, which is our primary application domain. The results of ViVaMBC at the SNP level are reported in Additional file 1.

The estimated frequencies of the true SNPs for the mixing proportions 1:200, 1:100, and 1:50 are presented in Table 3 for the existing methods. None of them were able to retrieve all 5 SNPs at a frequency of 0.5%. LoFreq could recover them at a frequency of 1% while the others still showed false-negative findings for the 1:100 mixtures. ViVaMBC, on the other hand, was able to discover both codon variants at a frequency of 0.5% and above (Table 1).

**Table 3:** **Sensitivity and specificity of competing methods in plasmid experiment**

	**LoFreq**			**V-Phaser2**			**ShoRAH**		
SNP (WT)	1:200	1:100	1:50	1:200	1:100	1:50	1:200	1:100	1:50
A (G)	/	1.03	2.41	0.59	1.06	2.37	/	/	2.22
G (C)	0.54	1.01	2.38	/	0.94	2.33	/	/	2.22
A (C)	0.66	1.03	2.16	/	/	/	0.44*	0.80*	1.78*
A (G)	0.48	0.91	2.10	0.52	1.04	2.11	0.44*	0.80*	1.78*
A (G)	/	0.89	2.05	0.48	/	2.07	/	/	1.28
*N*°false SNPs	3	5	2	19	32	24	4	1	1
Max Freq false SNPs	1.04	1.01	1.02	0.97	1.40	0.72	0.92*	0.5*	0.89

Frequency estimates of the true SNPs after applying the algorithms LoFreq, V-Phaser 2 and ShoRAH on the mixture of plasmids mixed at 1:200, 1:100 and 1:50. Two SNPs should be present in codon 36, while three SNPs are present in codon 155. In case of ShoRAH, the frequency is estimated from three overlapping windows, but often the variant is detected in two out of three windows (denoted with *). None of the methods seem to be able to retrieve all 5 SNPs at 0.5%. The bottom rows of the table report the total number of false SNPs over the whole NS3 region (543 bp long) together with their maximum frequency. The total number of false-positive findings is very low for all methods but their frequencies rise close to 1% which hamper the distinction of true SNPs from this false-positive findings.

**Figure 2:**
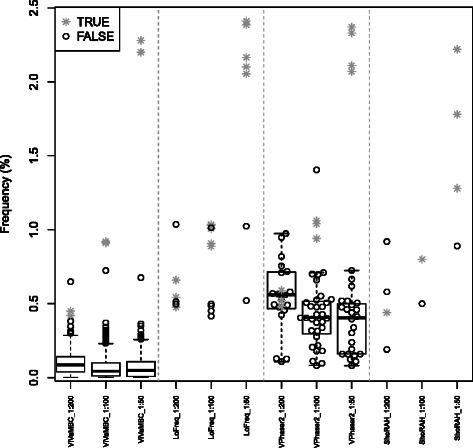
**Specificity comparison of ViVaMBC with LoFreq, V-phaser 2 and ShoRAH.** The frequencies of all minor variants discovered in the three mixtures 1:200, 1:100 and 1:50 are plotted for ViVaMBC, LoFreq, V-phaser 2 and ShoRAH. Note that these variants are at the codon level for ViVaMBC and at the SNP level for the other methods. The false positive variants are indicated with black dots and the true positives with gray crosses. It is clear that although far more false-positive findings are discovered with ViVaMBC, the distinction with the true positives is more apparent.

The total number of false discoveries over the whole NS3 region (181 codons of 3*b* *p* long) are reported at the bottom of Table 3 together with the maximum frequency of these false-positive findings. All methods seem to control the total number of false-positive findings much better than ViVaMBC, but the frequencies of these false-positive findings are close to 1% or even above and hamper the discovery of true variants with similar frequencies. Despite the higher number of false-positive findings discovered in ViVaMBC, a clear distinction between true- and false-positive findings can be made for frequencies around 1%. And with one exception, all false-positive findings fall below 0.4% (see Figure 2). So overall ViVaMBC has a higher sensitivity and specificity for the discovery of codon variants at frequencies above 0.5%.

V-phaser 2 and ShoRAH have add-on tools, V-profiler [27] (v1.0) and localVariants [28] (version january 8th 2014), respectively, to convert lists of SNPs to lists of codon variants which allows for direct comparison. LocalVariants is an unpublished tool which is still under development and until now we were unable to run it on our data. At this moment, it failed to define the reading frame based on the number of stop codons. V-Profiler is developed as an add-on tool for V-phaser and the output of V-phaser 2 must be converted to serve as an input for V-profiler. Both V-profiler and localVariants primarily focused on 454 data, and only shifted later to Illumina sequencing. The add-on tools are not fully converted yet, which makes the translation of the list of SNPs to a list of codon variants not straightforward. This illustrates the challenges of retaining linkage information between neighboring SNPs and the need for variant calling methods at the codon level.

### Clinical sample

**Figure 3:**
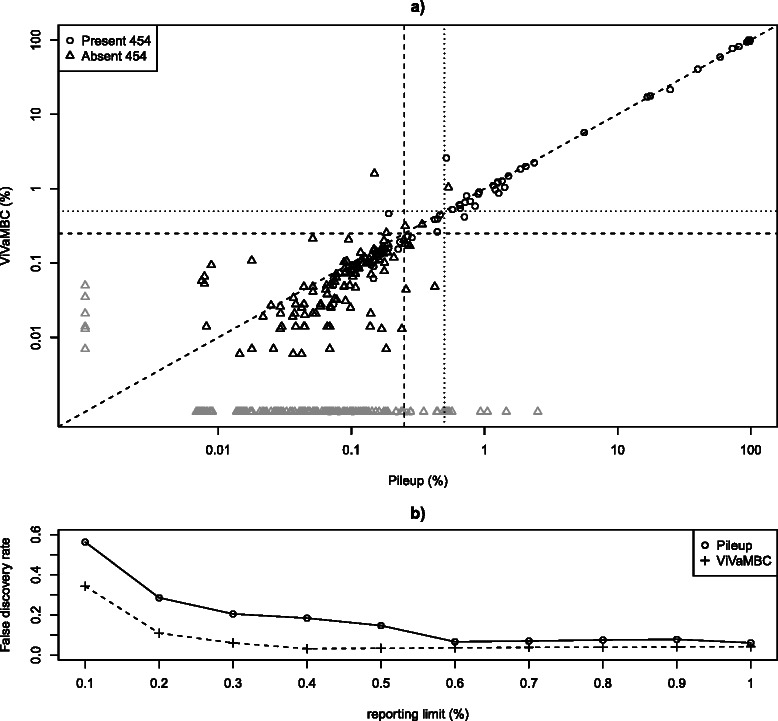
**Sensitivity and specificity comparison of ViVaMBC with pileup of a clinical HCV sample.** **a)** Comparison of the codon frequencies after piling up the data (x-axis) with the estimated frequencies of ViVaMBC (y-axis). Codons represented with triangles were absent after 454 sequencing on the same sample and hence assumed to be false-positive findings. Codons colored in grey are present in either one of the two methods. Frequencies of 0.5% and 0.25% are indicated with dotted and dashed lines respectively. Above 0.5% and even above 0.25% a good correlation is observed where a few false-positive findings are filtered out using ViVaMBC **b)** False discovery rates for both ViVaMBC and pileup are calculated with changing reporting limits. The FDR is higher and increases more rapidly for the pileup.

The application of ViVaMBC is illustrated here on a clinical HCV sample for which the NS3 amino acids 1 to 181 were sequenced with two sequencing platforms (454 and Illumina). The error prone GC-region was used for assessing the performance of ViVaMBC but we compared here the conclusions of the two platforms on the same sample. As 454 sequencing technology uses a different sequencing chemistry (see protocol in Thys K, Verhasselt P, Reumers J, Verbist BMP, Maes B, Aerssens J. Performance assessment of the Illumina massively parallel sequencing platform for deep sequencing analysis of viral minority variants, submitted) it typically results in another error profile. Variants not discovered with 454 can thus be assumed to originate from Illumina sequencing errors (and vice versa). In Figure 3a the estimated frequencies of the codons discovered by ViVaMBC are plotted against the corresponding frequencies of the pileup. Codons present in only one of the two methods are plotted in gray on their respective axis. Codons that were not present after piling up the 454 reads were indicated with triangles. Above 0.5% (dotted lines) a good correlation is observed between the two estimates. A few codons with frequencies above 0.5% in the pileup are not reported by ViVaMBC. These codons were also absent in 454 reads and can be considered as false-positive findings in the pileup. On the other hand, three codons showed a frequency above 1% with ViVaMBC while they had a lower frequency in the pileup, one of which was only present in 454. These codons might be false-positive findings called by ViVaMBC, however since it is a clinical sample, it is difficult to assess. Overall, ViVaMBC has a very good sensitivity; none of the true variants discovered with Pileup was missing.

The false discovery rate (FDR), calculated as the number of false-positive findings (codons not present in 454) divided by the total number of discovered codons is investigated for different reporting limits ranging from 0.1% to 1% for both ViVaMBC and pileup (Figure 3b). ViVaMBC has much lower FDR compared to pileup table for all reporting limits under investigation. While the FDR rapidly increases at low frequencies for the pileup, it remains stable for ViVaMBC up to a frequency of 0.4% before increasing, which is again in the frequency region where PCR errors start to occur as well. Moreover, the 454 experiment was limited in its detection due to the limited depth of coverage.

**Figure 4:**
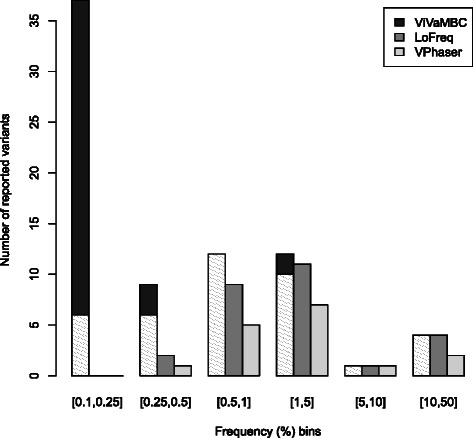
**Comparison of LoFreq and V-Phaser with ViVaMBC on clinical sample.** Barplot represents the number of reported variants (at SNP or codon level) by the different methodologies for different frequency bins. The bars are colored according to the method. The shaded region in the bars for ViVaMBC corresponds to the fraction of codons that were also discovered with 454.

Additionally, the three methods LoFreq v0.5.0 [10], V-Phaser 2 v2.0 [12], and ShoRAH v0.8 [5] were ran on the clinical sample. ShoRAH, however, crashed in the final stage of the analysis while running the snv.py script. Hence, Figure 4 only presents the comparison of the results of ViVaMBC with those of LoFreq and V-Phaser at SNP level using a barplot representing the number of reported variants at a particular frequency range. The shaded region in the bars for ViVaMBC corresponds to the fraction of codons that were also discovered with 454. Each of the codons reported both by ViVaMBC and 454, contains at least one SNP that should be detected by LoFreq and V-Phaser. V-Phaser, however, reports fewer variants in the majority of the bins, which indicates that it misses some true positives even at higher frequencies. LoFreq seems to perform better and detects all variants up to 1% but is less sensitive at lower frequencies. ViVaMBC probably reports two false positives in the frequency bin [ 1*%*−5*%*], these were also indicated in Figure 3a, but our method detects far more true positives especially in low-frequency ranges as compared to the other methodologies. The results confirm that codon variants with frequencies down to 0.5% can be reliably detected with ViVaMBC and that false positives start to appear at lower frequencies. Even down to 0.25% the proportion of false positives remains acceptable.

## Discussion

Many SNP calling tools have been described in the literature to correct sequencing errors. Most approaches are however tailored to call SNPs in human resequencing projects [29] where SNPs can only be either heterologous (50%) or homologous (100%). In viral deep sequencing projects, SNPs present in less than 1% of the reads are often of interest [30] making the correction much more challenging. Wilm et al. [10], among others, have shown that incorporating quality scores improves sensitivity without loss of specificity. The comparison with existing tools showed, however, that issues with the detection of low frequency variants with a frequency below 1% in viral populations remains largely unsolved by the currently available methods. ViVaMBC embeds quality scores and the second best base calls within a model-based clustering approach. The method enables an increase in sensitivity for variants with frequencies below 1%, while retaining good specificity above 0.4%. When no second best base calls are available, ViVaMBC still shows an improved sensitivity in comparison with the existing methodologies. Although the potential of the second best base calls seemed very promising in the data exploration phase, the additional computational cost of running the offline base caller is not warranted for our specific application. At frequencies below 0.4% we start to see errors where some of them are presumed as being incorporated during sample and library preparation. These artificial mutations cannot be identified as errors because the base substitutions are passed to all sequences of the cluster on the flow cell. Hence, these sample and library preparation errors form the limit for detection since only sequencing errors can be corrected with ViVaMBC. To obtain excellent sensitivity and specificity, samples need to be sequenced deep enough. When coverage falls below 25,000 the number of false-positive findings increases and the frequency estimates become biased. Furthermore, ViVaMBC is one of the first tools that calls variants at the codon level, which is particularly of interest in virology applications where drug-target regions are investigated for resistance-associated amino acid mutations.

The current version of ViVaMBC assumes that each of the *n* reads covers the entire window of *m* nucleotides. In practice, many reads cover only partially the window. Although these reads are currently ignored by our method it has a fairly low impact on the results as variant calling is done at the codon level *m*=3. Ignoring reads can become problematic when larger window sizes *m* are of interest. If one assumes missingness completely at random, the likelihood approach could be continued with the observed data only. The method only has to be adapted to work with unbalanced data; not all reads will have the same length *m*. Let *v*_*il*_ denote an indicator which is *v*_*il*_=1 if read *i* has a call at position *l* and zero otherwise. The density *f*_*j*_ in (1) and (2) become

(9) $$ \begin{array}{rr} &f_{j}(\mathbf{r}_{i},\mathbf{s}_{i}) = \prod\limits_{l=1}^{m} f_{j}(r_{il},s_{il})^{v_{il}}\\ &= \prod\limits_{l=1}^{m} \left[\theta_{ril}^{I({r_{il}=h_{jl}})} \theta_{sil}^{I({s_{il}=h_{jl}})} \theta_{oil}^{(1-I({r_{il}=h_{jl}}))(1-I({s_{il}=h_{jl}}))}\right]^{v_{il}}. \end{array}  $$

Subsequently, the complete data log-likelihood (6) becomes

(10) $${} \begin{aligned} l&= \log L = \sum\limits_{i=1}^{n} \sum\limits_{j=1}^{k} z_{ij} \left\{ \log\tau_{j} + \sum\limits_{l=1}^{m} v_{il} \left[ I^{(r)}_{ijl} \log\theta_{ril} \right.\right.\\ &\qquad +\left. \left. I^{(s)}_{ijl} \log\theta_{sil} + \left(1-I^{(r)}_{ijl}\right)\left(1-I^{(s)}_{ijl}\right)\log\theta_{oil}\right] \vphantom{\left\{ \log\tau_{j} + \sum\limits_{l=1}^{m} v_{il} \left[ I^{(r)}_{ijl} \log\theta_{ril} \right.\right.}\right\}. \end{aligned}  $$

We successfully ran ViVaMBC for a number of HCV-clinical samples where the whole NS3 region is assessed. Investigation of the reported codons will help us to discover mutations associated to resistance against protease inhibitors and to establish the clinical relevance of resistance associated mutations [31]. While ViVaMBC is especially developed for virology applications it might be also applicable in targeted sequencing of cancer associated genes where one wants to uncover the tumor-population heterogeneity. These targeted cancer panels investigate again coding regions, hence working at the codon level makes absolutely sense here.

## Conclusion

ViVaMBC is proposed for identifying variants at the codon level within a viral population using Illumina sequencing. The parameters *τ*_*j*_ and **h**_*j*_ define the local viral population and are inferred given the observed data. We demonstrated here a superb sensitivity of ViVaMBC while keeping the frequencies of the false-positive findings below 0.4% when an average coverage of 25,000 is reached. The strength of the method lies in modeling the error probabilities, based on the quality scores, which enables to correct a large fraction of the mismatch bases incorporated during the sequencing process. When no second best base calls are available, ViVaMBC can be run without them while it still provides an optimal sensitivity when reporting limits of 0.5% are applied. The technical constraints like PCR errors start to form the bottleneck for low-frequency variant detection.

## Additional file

Additional file 1
**Supplementary information.** Contains additional information regarding the data and the workflow as well as a link to the R-code.
