# Supplementary material for: ViVaMBC: estimating viral sequence variation in complex populations from illumina deep-sequencing data using model-based clustering
Source: BMC Bioinformatics. 2015 Feb 22;16:59. doi: 10.1186/s12859-015-0458-7 (PMC4369097; doi:10.1186/s12859-015-0458-7)
Supplement: Additional file 1 — Supplementary information. Contains additional information regarding the data and the workflow as well as a link to the R-code. [file 12859_2015_458_MOESM1_ESM.zip › supp1.pdf]

RESEARCH

Additional file 1. ViVaMBC: estimating Viral sequence Variation in complex populations from Illumina deep-sequencing data using Model-Based Clustering.

Bie Verbist[1], Lieven Clement[2], Joke Reumers[3], Kim Thys[3], Alexander Vapirev[3,4], Willem Talloen[3], Yves Wetzels[3], Joris Meys[1], Jeroen Aerssens[3], Luc Bijnens[3] and Olivier Thas[1,5*]

*Correspondence: Olivier.Thas@ugent.be
[1]Department of Mathematical Modeling, Statistics and Bioinformatics, Ghent University, Coupure Links 653, 9000 Gent, Belgium
Full list of author information is available at the end of the article

Sample Preparation

The preparation of the HCV-NS3 plasmids as well as the HCV clinical samples can be found in Thys, K et al, Journal of Virological methods. The sequencing protocols for Illumina and 454 are also described in the above mentioned paper.

Workflow

1 Off-line base calling with the option of second best base call:
```
bustard.py --CIF <directory with intensities> --make --with-second-call
--with-qseq --keep-dif-files
```
2 Demultiplex:
```
configureBclToFastq.pl --input-dir <input directory>  --output-dir
<output directory>
```
3 Alignment with for instance BWA, creating a sam file

4 Convert second best base qseq files to fastq with fastqconverter from Casava:
```
FastqConverter --in <inputfile> --out <outfile>
```
5 Add second best base call to sam, E2 and U2 tags are standard foreseen in sam to be filled by second best base calls and there quality scores: own Perl script submitted at sourceforge [http://sourceforge.net/p/vivambc/code/ci/master/tree/]

6 Convert sam to sorted bam with samtools:
```
samtools view -b -t <fasta of reference> <input sam file> | samtools
sort - <name of output>
```
7 Create heading with picard in order to be able to perform the next step:
```
java -jar /opt/picard-tools-1.86/AddOrReplaceReadGroups.jar I=<inputfile>
 O=<outputfile> LB=none PL=illumina PU=none SM=none
```
8 Change bam positions using clipreads of GATK:
```
java -jar /opt/GenomeAnalysisTK-2.3-9/GenomeAnalysisTK.jar -T ClipReads
-I <input bam file> -o <output bam file > -R <input ref file> -CR
HARDCLIP\_BASES
```
9 Run R-script to perform model based clustering

R-code

The R-code together with the perl scripts are available under code at:

http://sourceforge.net/p/vivambc/code/ci/master/tree/

The R-code is parallelized which makes it possible to run each codon position on a separate core in order to speed up. The following command can be used:

```
mpirun -np <number of nodes> Rscript ViVaMBC.R.
```

The output is a codonTable.txt file where the position, the codons and their frequencies are reported. The code is tested on Amazon Web Services (AWS). For a region covering 181 windows the code runs for approximately 12 hours when a server with 16 cores and 60GB of RAM is used. Without parallelization it would take more than 7 days to obtain the results. Further optimalization of the R-code is most probably possible.

Error Correction by second best base calling

The amount of errors that could be corrected by second best base calls is determined using the mixture of plasmids. These mixtures have two variant positions, position 36 (GTC (consensus) $\rightarrow$ ATG) and 155 (CGG (consensus) $\rightarrow$ AAA). All codons that are different from the two possible codons at each variant position are considered as error. For each false positive we check where the error occurs; which nucleotide or nucleotides within the codon differs from the ones in the true codons. In the next step, it is tested if the replacement of the error by the second best base call delivers one of the true codons. This procedure is repeated for the 4 mixing proportions, 1:200, 1:100, 1:50, and 1:10. In total 70% of the errors could be corrected by the second best base call as presented in Additional Figure 1. The individual percentages for each codon position and each mixing proportion is shown in Table 1.

Pileup

The results of ViVaMBC are compared with the codons present in the raw data after trimming (Table 2, main paper). The trimming is done by removing all bases, soft clipped by the alignment tool which is indicated in the CIGAR with S. These bases are most likely errors since no translocations or big deletions are expected in the NS3 region. After trimming, all codons present in the data will be tabulated for each codon position. In the main paper this approach will be called pileup (in analogy with mpileup of samtools).

ViVaMBC at the SNP level

ViVaMBC has been optimized for $m = 3$ to retain linkage information between single nucleotide polymorphism, which allows for an immediate biological interpretation. Nonetheless, the algorithm can be applied with different window sizes m.

We have run ViVaMBC with window size $m = 1$ to call SNPs on the mixture of plasmids, in analogy with the existing methods. The estimated frequencies of the 5 known SNPs are reported in Table 2. All variants could be retrieved with frequencies close to the mixing proportions. All other variants, besides the 5 SNPs, are assumed to be false-positive findings. ViVaMBC at the SNP level reports more false-positive findings compared with the existing methodologies similar to ViVaMBC at codon level (Table 2). However, their frequencies remain well below 0.35%. Only one outlier was observed at a frequency of 0.65% for the mixing proportion 1:100 (Additional Figure 3). Overall ViVaMBC has a higher sensitivity and specificity for the discovery of SNPs down to a frequency of 0.5% in comparison with the other methods.

Contribution of second best base calls

ViVaMBC clusters variants based on the error probabilities of both the first and the second best base call. These second best base calls must be retrieved during base-calling. However, in some cases these second best base calls might be difficult to get, for instance sequencing providers perform often RTA and provide the best base calls only. Therefore, the performance of ViVaMBC is checked if only the best base calls are available. Equation 3 simplifies than to a simple logit model where c equals r instead of a multinomial logit model. Sensitivity and specificity is again investigated using the mixture of plasmids. The results are displayed in Table 3. The estimated frequencies of the two real variants are close to the mixing proportions for the four different mixes. The number of reported variants over the whole NS3 region is displayed in the fourth column of Table 3 together with the maximum frequency of the false-positive findings among the reported codons. For the plasmid mixtures, each codon differing from the wild type other than the spiked-in variant is considered to be an error. Hence, exclusion of second best base calls seems to have an increase in the specificity of the method without losing the sensitivity. However, some of the sequence differences at low-frequencies are expected to be real as they might originate from errors introduced during plasmid preparation. Hence, the increase in specificity is most probably a trade-off with the sensitivity for the very low-frequency variants.

Additionally, the influence of coverage depth on the accuracy of the frequency estimates is investigated using the plasmid data, mixed 1:200, at codon position 155. A similar experimental setup was performed as described in the main paper. The frequencies of the variants for this position for each of the 90 re-sampled datasets are plotted in Additional Figure 4. The true codon variant AAA (green dots) was detected in all datasets. Averages frequency estimates over the 10 repeats are indicated with green triangles. The frequency estimate based on the error probabilities of best and second best base calls on the full dataset is indicated with a horizontal dashed line. In general, the frequency estimates are slightly underestimated. The number of false-positive findings is again much lower in comparison with ViVaMBC where the error probabilities of the second best base calls are taken into account. Although this might hint to an increased specificity, it is possible that it actually implies a decreased sensitivity as suggested above.

To investigate this further the method is applied on the GC-rich region of the clinical HCV sample used in the main paper. The ViVaMBC results with and without second best base calls are plotted on the y- and x-axis respectively in Additional Figure 5. Codons that are exclusively reported with one of the methods are displayed in gray on the corresponding axis. The variants that were not present after 454 sequencing are displayed with triangles. Above 0.5% the two methods are in agreement, with slightly lower frequency estimates when omitting the second best base calls. Further, inclusion of the second best base calls in the model based clustering reveals more variants (similar to the results of the HCV plasmid) and sixteen of them are reported with the 454 experiment with frequencies up to 0.3%. Note, that the number of missed discoveries is probably higher since the 454 experiment was not sequenced deep enough to reveal frequencies below 0.05%. This suggests that some sensitivity is lost when second best base calls are omitted. However, both

ViVaMBC implementations give very similar results when a reporting limit of 0.5% is applied.

Figure 1 Error correction by second best base calls Pie charts where the total pie represents the number of errors observed. 70% of them could be corrected by the second best base call, while the other 30% remain error.

Figure 2 Frequency distribution of the false-positive findings for the 4 mixing proportions after pileup and ViVaMBC The raw data include some error induced variants with frequencies above 0.5% and even 1% which will hamper the discovery of the true low-frequency variants at position 36 and 155 (black boxplots). After applying ViVaMBC the frequencies of these false-positive findingss only reach 0.4% with one exception over the 4 mixing proportions (grey boxplots).

Figure 3 ViVaMBC at SNP level Boxplots of the frequencies of all minor variants discovered in the three mixtures 1:200, 1:100 and 1:50 are plotted for ViVaMBC at SNP level. The true positives are indicated with gray crosses.

Figure 4 Influence of coverage depth on the estimation of τ_j when ViVaMBC is solely based on the error probabilities of the best base calls. Datasets with lower coverages are generated by random sampling a fraction (f=0.1, 0.2, . . . , 0.8 ,0.9) of the reads from the original dataset. Ten datasets were generated for each fraction f resulting in 90 datasets. The reported variants for all re-sampled datasets were plotted and colored according to the discovered codon. The green dots indicate the true variant and the few others are false-positive findings. The average frequency of the true variant (averaged over the ten random samples) is indicated with triangles. The dotted line is the true frequency as estimated from the original dataset when the error probabilities of the second best base calls are taken into account.

Figure 5 Impact of the second best base call error probability on ViVaMBC The frequency estimates of the codons revealed by ViVaMBC with and without second best base calls are plotted on the y-axis and x-axis respectively. Codons that are exclusively reported by one of the methods are plotted on the respective axis in gray. Codons represented with triangles were absent after 454 sequencing on the same sample and hence assumed to be false-positive findings. The reporting limits of 0.5% and 1% are displayed with dashed lines.

Table 1 Percentage of errors that could be corrected by second best base calls for each variant position and for each mixing proportion.

Pos	1:200	1:100	1:50	1:10
36	72.5	69.6	71.0	68.2
155	73.1	73.1	72.5	65.6

Author details
[1]Department of Mathematical Modeling, Statistics and Bioinformatics, Ghent University, Coupure Links 653, 9000 Gent, Belgium. [2]Department of Applied Mathematics, Informatics and Statistics, Ghent University, Krijgslaan 281 S9, 9000 Gent, Belgium. [3]Janssen Pharmaceutica R&D, Turnhoutseweg 30, 2340 Beerse, Belgium. [4]ExaScience Life Lab, Kapeldreef 75, 3001 Leuven, Belgium. [5]University of Wollongong, National Institute for Applied Statistics Research Australia (NIASRA), School of Mathematics and Applied Statistics, NSW 2522, Australia.

References
1. Thys, K., Verhasselt, P., Reumers, J., Verbist, B.M.P., Maes, B., Aerssens, J.: **Performance Assessment of the Illumina Massively Parallel Sequencing Platform for Deep Sequencing Analysis of Viral Minority Variants.** *Journal of Virological Methods* 2014, **submitted**.

Table 2 Sensitivity and specificity of ViVaMBC at the SNP level. Frequency estimates of the true SNPs are close to the mixing proportions for all 3 mixes under investigation (1:200, 1:100 and 1:50). The bottom rows of the table report the total number of false SNPs over the whole NS3 region (543 bp long) together with their maximum frequency.

		ViVaMBC		
	SNP (WT)	1:200	1:100	1:50
36	A (G)	0.49	0.93	2.23
	T (T)			
	G (C)	0.43	0.88	2.18
155	A (C)	0.49	0.89	2.15
	A (G)	0.45	0.89	2.20
	A (G)	0.43	0.87	2.14
Number of false SNPs		132	139	209
Max Freq of false SNPs		0.32	0.65	0.34

Table 3 Sensitivity and specificity of ViVaMBC in plasmid experiment when only the error probabilities of the best base calls are incorporated in the model. The estimated frequencies of the variants at codon position 36 and 155 for the four different mixing proportions are displayed together with the number of reported codons and the maximum frequency of the false-positive findings among them.

Mixing Prop	36 ATG (%)	155 AAA (%)	N° Codons	max noise freq (%)
1:200	0.44	0.40	289	0.52
1:100	0.89	0.84	291	0.51
1:50	2.20	2.16	301	0.57
1:10	10.82	9.89	253	0.38
