# Supplementary figures and images for: ViVaMBC: estimating viral sequence variation in complex populations from illumina deep-sequencing data using model-based clustering
Source: BMC Bioinformatics. 2015 Feb 22;16:59. doi: 10.1186/s12859-015-0458-7 (PMC4369097; doi:10.1186/s12859-015-0458-7)

## Correction by Second Best Base Calls

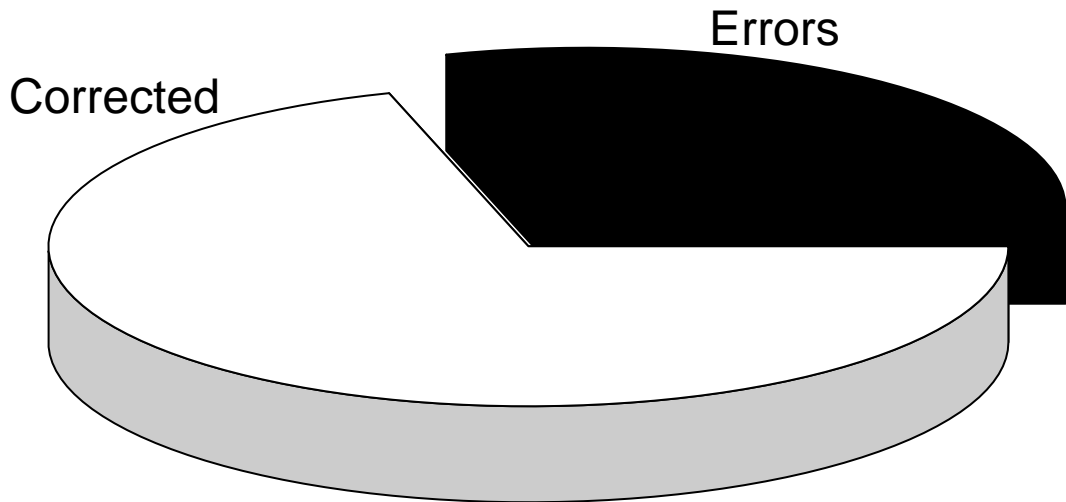

Supplement: Additional file 1 — Supplementary information. Contains additional information regarding the data and the workflow as well as a link to the R-code. [file 12859_2015_458_MOESM1_ESM.zip › Figure_S1.pdf]

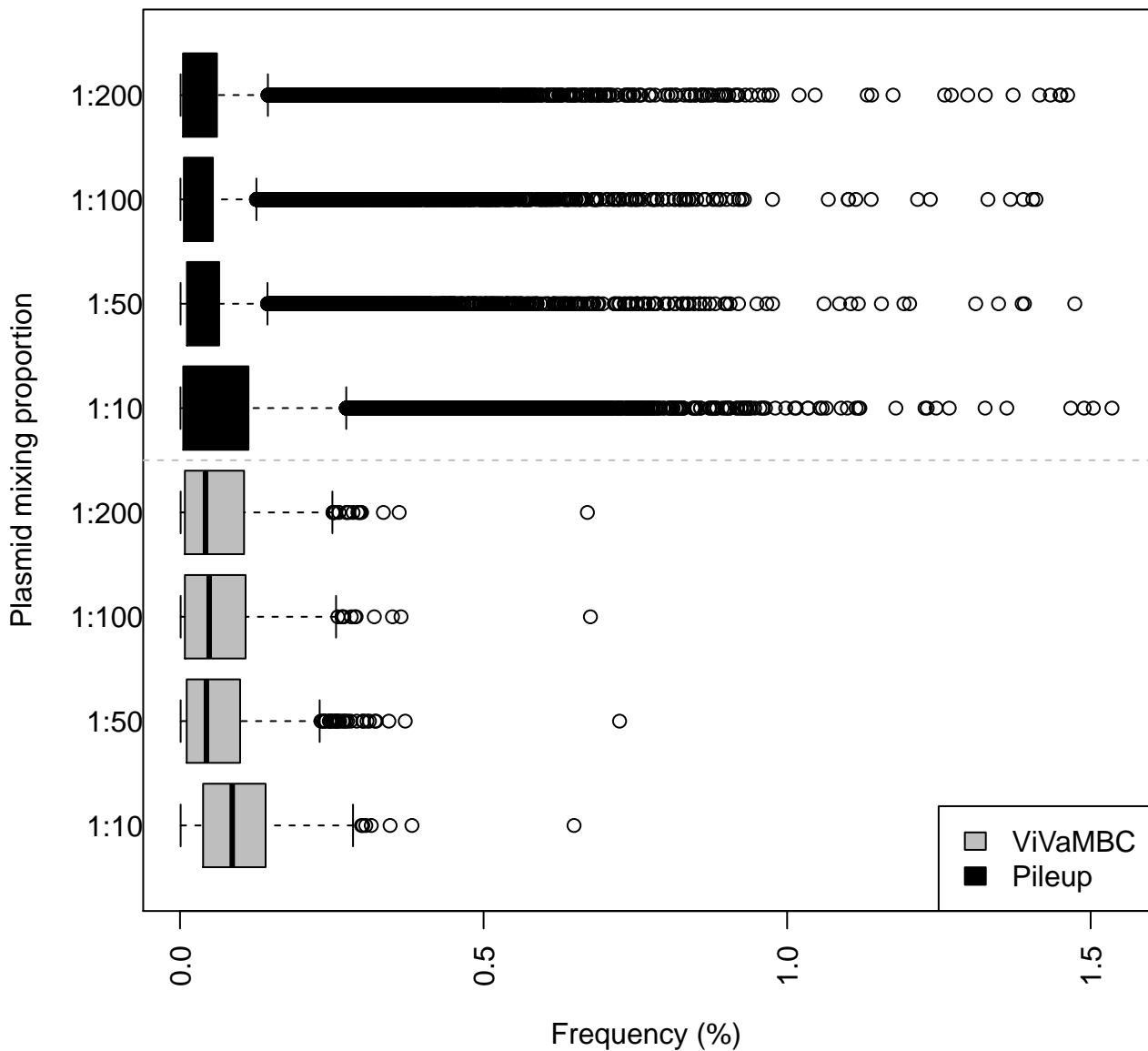

Supplement: Additional file 1 — Supplementary information. Contains additional information regarding the data and the workflow as well as a link to the R-code. [file 12859_2015_458_MOESM1_ESM.zip › Figure_S2.pdf]

# SNP level

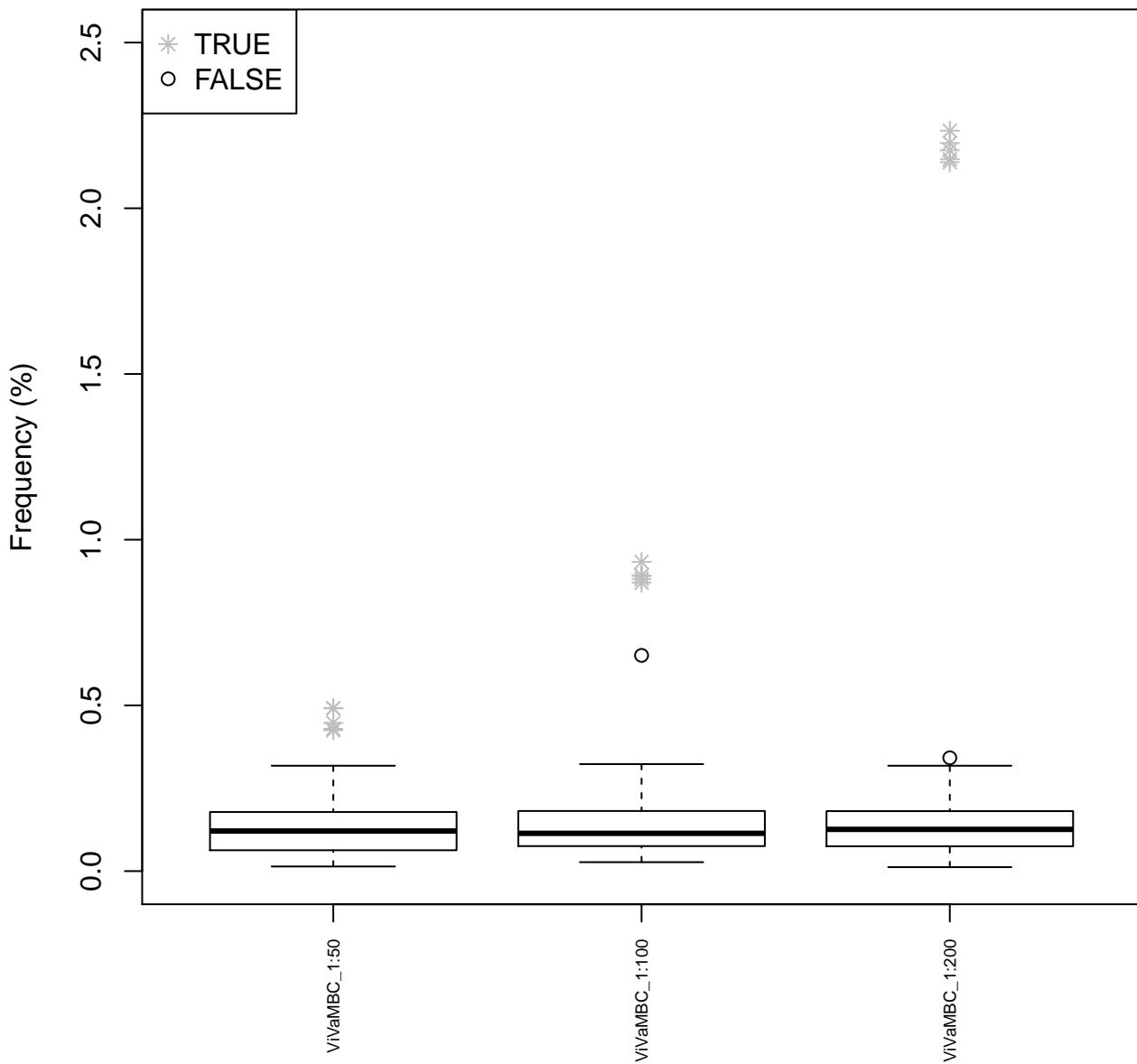

Supplement: Additional file 1 — Supplementary information. Contains additional information regarding the data and the workflow as well as a link to the R-code. [file 12859_2015_458_MOESM1_ESM.zip › Figure_S3.pdf]

# Codon 155

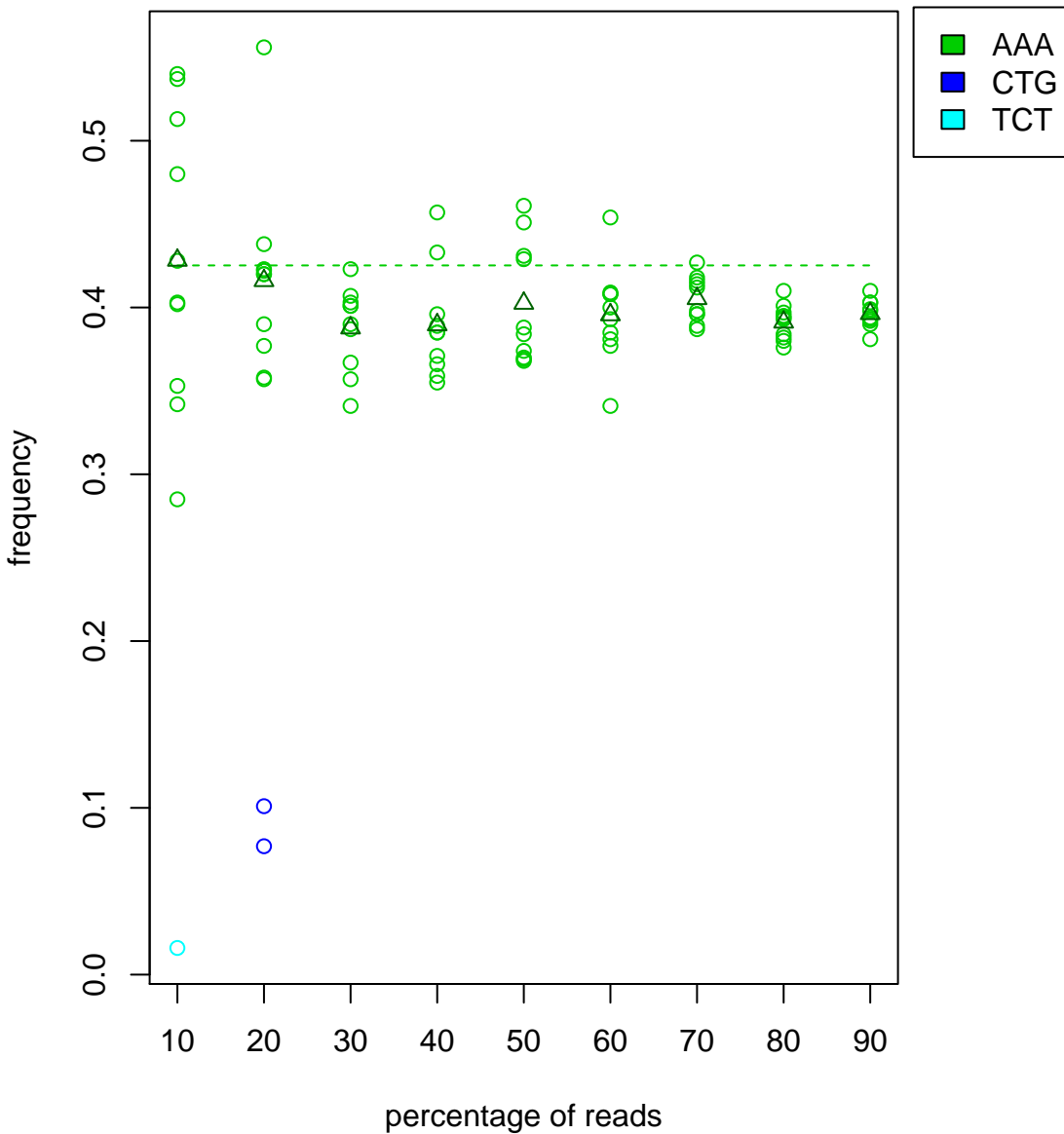

Supplement: Additional file 1 — Supplementary information. Contains additional information regarding the data and the workflow as well as a link to the R-code. [file 12859_2015_458_MOESM1_ESM.zip › Figure_S4.pdf]

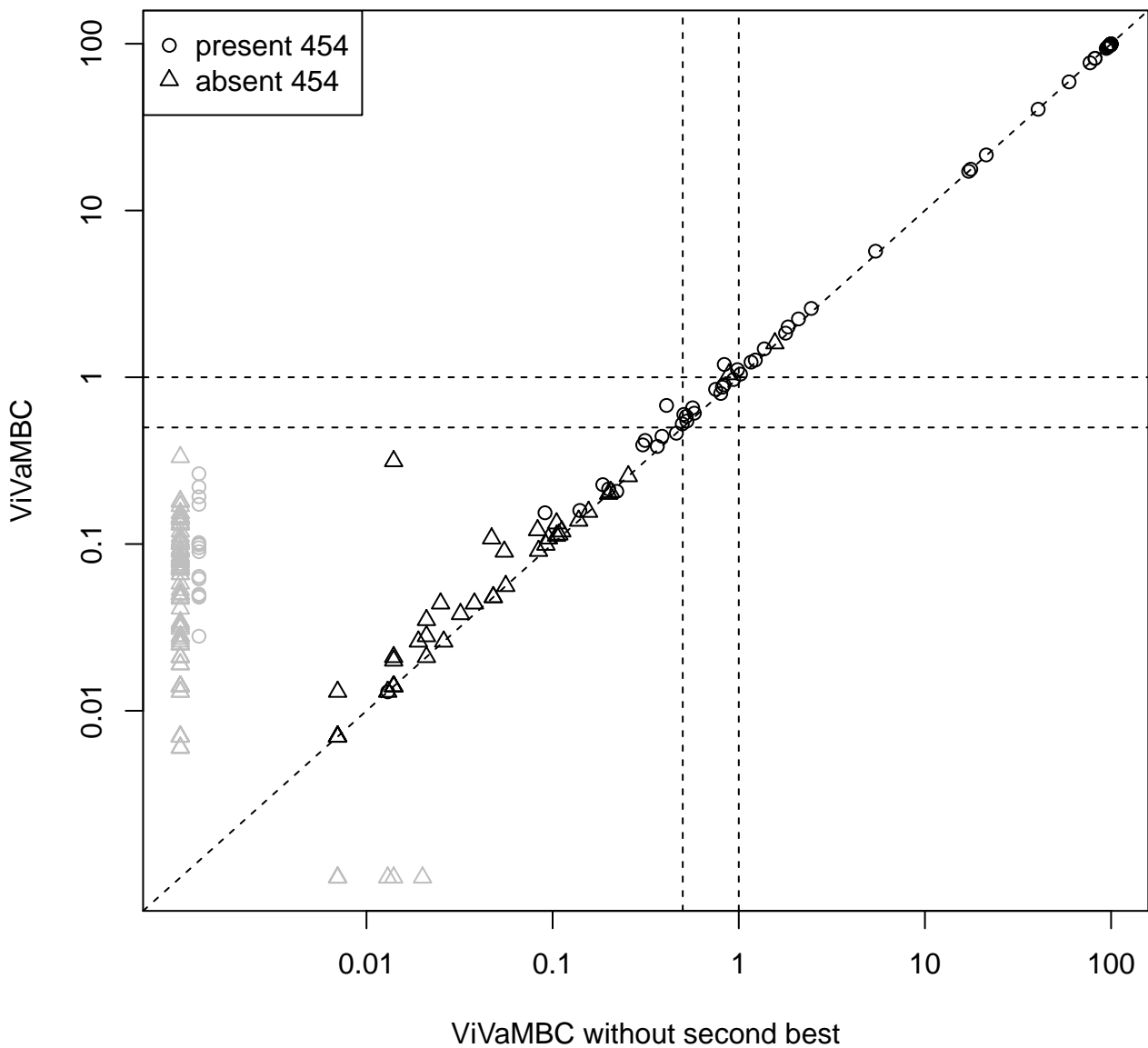

Supplement: Additional file 1 — Supplementary information. Contains additional information regarding the data and the workflow as well as a link to the R-code. [file 12859_2015_458_MOESM1_ESM.zip › Figure_S5.pdf]
